# Differentiating irritable mood and disruptive behavior in adults

**DOI:** 10.1590/2237-6089-2019-0078

**Published:** 2020-11-17

**Authors:** Ana Cláudia U. Knackfuss, Ellen Leibenluft, Melissa A. Brotman, Érico de Moura Silveira, André Simioni, Lorenna S. Teixeira, Luciana Gerchmann, Adam Fijtman, Andrea R. Trasel, Daniela Sperotto, Arthur G. Manfro, Flávio Kapczinski, Márcia K. Sant’Anna, Giovanni A. Salum

**Affiliations:** 1 Hospital de Clínicas de Porto Alegre Departamento de Psiquiatria Universidade Federal do Rio Grande do Sul Porto AlegreRS Brazil Seção de Afeto Negativo e Processos Socias, Hospital de Clínicas de Porto Alegre, Departamento de Psiquiatria, Universidade Federal do Rio Grande do Sul (UFRGS), Porto Alegre, RS, Brazil.; 2 Section on Mood Dysregulation and Neuroscience Intramural Research Program National Institute of Mental Health BethesdaMD USA Section on Mood Dysregulation and Neuroscience, Intramural Research Program, National Institute of Mental Health, Bethesda, MD, USA.; 3 Hospital de Clíncias de Porto Alegre Departamento de Psiquiatria Universidade Federal do Rio Grande do Sul Porto AlegreRS Brazil Programa de Transtorno Bipolar, Laboratório de Psiquiatria Molecular, Hospital de Clíncias de Porto Alegre, Departamento de Psiquiatria, Universidade Federal do Rio Grande do Sul (UFRGS), Porto Alegre, RS, Brazil.

**Keywords:** Irritable mood, factor analysis, behavior disorders

## Abstract

**Introduction:**

Irritability has both mood and behavioral manifestations. These frequently co-occur, and it is unclear to what extent they are dissociable domains. We used confirmatory factor analysis and external validators to investigate the independence of mood and behavioral components of irritability.

**Methods:**

The sample comprised 246 patients (mean age 45 years; 63% female) from four outpatient programs (depression, anxiety, bipolar, and schizophrenia) at a tertiary hospital. A clinical instrument rated by trained clinicians was specifically designed to capture irritable mood and disruptive behavior dimensionally, as well as current categorical diagnoses i.e., intermittent explosive disorder (IED); oppositional defiant disorder (ODD); and an adaptation to diagnose disruptive mood dysregulation disorder (DMDD) in adults. Confirmatory factor analysis (CFA) was used to test the best fitting irritability models and regression analyses were used to investigate associations with external validators.

**Results:**

Irritable mood and disruptive behavior were both frequent, but diagnoses of disruptive syndromes were rare (IED, 8%; ODD, 2%; DMDD, 2%). A correlated model with two dimensions, and a bifactor model with one general dimension and two specific dimensions (mood and behavior) both had good fit indices. The correlated model had root mean square error of approximation (RMSEA) = 0.077, with 90% confidence interval (90%CI) = 0.071-0.083; comparative fit index (CFI) = 0.99; and Tucker-Lewis index (TLI) = 0.99, while the bifactor model had RMSEA = 0.041; CFI = 0.99; and TLI = 0.99 respectively). In the bifactor model, external validity for differentiation of the mood and behavioral components of irritability was also supported by associations between irritable mood and impairment and clinical measures of depression and mania, which were not associated with disruptive behavior.

**Conclusions:**

Psychometric and external validity data suggest both overlapping and specific features of the mood vs. disruptive behavior dimensions of irritability.

## Introduction

Irritability can be defined as elevated proneness to anger relative to peers.^1 , 2^ As a symptom, it is present in several psychiatric disorders, including major depressive disorder (MDD), generalized anxiety disorder (GAD), and others.^3 - 7^ Irritability is also the core characteristic of three diagnoses in the 5th edition of the Diagnostic and Statistical Manual of Mental Disorders (DSM-5): intermittent explosive disorder (IED), oppositional defiant disorder (ODD), and disruptive mood dysregulation disorder (DMDD) – all of which require significant distress and impairment.^8^ IED is characterized by presence of disruptive behaviors (e.g., extreme temper outbursts, aggression), whereas ODD and DMDD are characterized by both disruptive behaviors and irritable mood, i.e., persistent anger, including sullen nonverbal behaviors, and reports of being annoyed over many days.^1^ Although irritable mood and behavior frequently co-occur, it is unclear whether these components of irritability can be measured separately and constitute distinct dimensions. Beyond the implications for the conceptualization of irritability in the adult population, if irritability has distinct components, this raises the possibility of tailoring interventions to target specific components of irritability. While research has begun to examine this question in youth,^9^ data in adults are lacking. If mood and behavioral aspects of irritability are distinct, this could also have important implications for research about etiology and therapies, which might differ for each of these dimensions. Better understanding of the phenomenology of irritability might allow for advances in classification of such symptoms, improve our current nosology and, perhaps, leverage future research to provide better care for patients with irritability. Therefore, the purpose of this study is to investigate distinctions between the mood and behavioral components of irritability in clinically referred adults.

One way of investigating whether behavior and mood are distinct constructs is to use confirmatory factor analysis (CFA). CFA starts from the assumption that indicators (i.e., symptoms) are initiated by non-observable latent constructs. By examining variance among those symptoms, theoretical models can be tested to decide which model is best suited to describing the symptom correlation patterns observed in a specific dataset. We tested three potential models. In the first, irritability is conceptualized as a single construct, i.e., all the variance of irritability symptoms is due to either a single latent factor or measurement error. The second model assumes that there are two correlated factors (mood and behavior), i.e., two separate, correlated sources of variance. Lastly, we tested a bifactor model, which assumes both shared and distinct sources of variance between irritable mood and disruptive behavior. CFA results can be complemented by examining correlations between the latent factors mood and behavior and external validators (i.e. measures from other instruments).

The adult literature on irritability has mainly focused on behavioral manifestations of irritability, conceptualized as reactive aggression.^10 , 11^ Aggression, defined as a behavior intended to harm another, is commonly divided into proactive and reactive aggression.^12^ Proactive, or instrumental, aggression is designed to achieve a goal (e.g., gain social status or a job promotion), while reactive aggression (also called emotional or hostile aggression) occurs in response to frustrating or threatening events. However, previous studies in the adult irritability literature did not focus on psychometrics, such that neither specific associations between reactive aggression and irritable mood, nor the extent to which these constructs are distinct could be directly investigated.

In contrast to the adult psychiatric literature, child psychiatry research demonstrates the importance of studying both behavioral and mood manifestations of irritability in depth.^1 , 13 , 14^ Research in children indicates that irritability is closely related to affective disorders such as anxiety and depression, in terms of longitudinal^15^ and genetic^16^ associations, as well as some shared cognitive mechanisms, such as biases towards threats.^17 , 18^ Thus, the mood and behavioral components of irritability might be alternative manifestations of the same pathophysiological mechanism. Alternatively, these associations could be driven by a shared affective component that is common to anxiety, depression, and anger, but which excludes the behavioral components of irritability. The latter hypothesis is only plausible if the mood and behavioral components of irritability are indeed distinct constructs, a hypothesis that is examined in this paper.

Here, we investigate the independence of the mood and behavioral components of irritability in a sample of adults with severe mental disorders, using confirmatory factor analysis and correlations with external validators. We hypothesize that a clinical interview specifically designed to probe mood and behavioral aspects of irritability will provide a means of investigating these two related dimensions. Consistent with this, measures of external validity will identify some specific associations with each of these two components of irritability.

## Methods

### Sample

The sample consisted of 246 patients, recruited from four outpatient programs at the Hospital de Clínicas de Porto Alegre (bipolar, n = 68; depression, n = 55; anxiety, n = 55; and schizophrenia, n = 68). Outpatient programs were selected by convenience and included most of the high-order adult psychiatric disorders. Data were collected from March to May of 2015 in the bipolar program, from June to September of 2015 in the schizophrenia program, from October of 2015 to January 2016 in the anxiety program, and from February to June of 2016 in the depression program. All patients attending the outpatient programs were invited to participate. The inclusion criterion was the ability to complete the study questionnaires. Patients were not included if they lacked the ability to read or write and/or were not able to adequately complete the questionnaires. Due to a simultaneous study investigating biomarkers, we excluded patients with inflammatory conditions, autoimmune disease, history of acute infections, drug and alcohol use disorders (except cigarettes and coffee), current pregnancy or breastfeeding, and organic mental illness (dementia, epilepsy, stroke). Individuals were recruited in the outpatient clinics waiting room, before their doctor’s appointments. In total, 944 patients were invited to participate, 254 refused, 71 failed to complete the study protocol, and 373 did not met the inclusion criteria or were excluded (26% for being unable to read or write, 16% for diabetes, 7% for epileptic seizures, 7% for HIV infection, 7% for hepatitis, and less than 4% for all other reasons). Therefore, 246 were included in the current analysis. All patients signed informed consent. The Hospital de Clínicas de Porto Alegre institutional review board approved the study.

### Procedures

Patients from each outpatient program were interviewed in two sessions. The first included administration of instruments by trained psychologists and medical students, including characterization of socioeconomic status and a self-report questionnaire, the Mood Disruptive Scale (MOODS), lasting ~30 minutes. The second session was a detailed clinical interview with two trained psychiatrists (L.S.T.M and E.M.S), which lasted around 40 minutes and included all of the clinical instruments described below, including the Mood Disruptive Diagnostic Instrument (MOODS-I).

### Instruments

#### The Mood Disruptive Scale (MOODS)

The MOODS is a self-rated questionnaire with 56 questions with Likert-type response scales covering the previous seven days, designed to measure levels of disruptive mood and behavior. It includes four main sections: disruptive behavior (temper outbursts), defiant and vindictive behavior, irritable mood (angry mood), and irritability-related impairment. Since the main aim of this study is to investigate distinctions between irritable mood and disruptive behavior, the defiant and vindictive behavior section was not analyzed. The disruptive behavior section consists of 17 questions evaluating behaviors during anger outbursts, including verbal and physical aggression, as well as anger directed towards objects and animals. Responses are distributed along a Likert scale assessing intensity with the following options: never, rarely, some days of the week, most days of the week, every day of the week, and many times each day. The irritable mood section is composed of 10 questions reflecting words commonly used to describe irritable mood. Responses are selected from an intensity scale with the following options: not at all, a little, moderately, a lot, and extremely. Finally, the impairment section includes 8 questions about the consequences of aberrant mood and behavior on overall functioning with family, friends, relationships, work, school, and the criminal justice system. Response options are distributed along an intensity scale with the following options: not at all, a little, a lot, and extremely. The MOODS self-rated data were used in the CFA to assess model fit.

#### Mood Disruptive Diagnostic Instrument (MOODS-I)

The MOODS-I is a clinician-rated, structured interview with several Likert-type scale questions which diagnoses current IED, ODD, and DMDD in adults. The instrument is a hybrid of (1) a structured interview with multiple-choice questions based on the DSM-5 to allow use of a computerized algorithm to assign diagnoses; and (2) a clinical judgment section rated by a trained clinician on the basis of the answers to the multiple-choice questions. The structured interview section includes detailed assessment of disruptive behavior, headstrong and vindictive behaviors, disruptive mood, and impairment. As in the MOODS, the MOODS-I includes several items assessing frequency and intensity of common behaviors occurring during a temper outburst (e.g. “In the past 12 months how often have you lost your temper?”); common terms designed to define the intensity of disruptive mood (e.g. “On a typical day in the past 12 months, for how long were you angry?”); detailed assessment of the consequences of irritability (e.g. “Do the irritability, defiance, or temper outbursts cause problems in you romantic life?”); and exclusionary criteria consistent with DSM-5. The clinical judgment section requires the clinician to rate each DSM-5 criterion answered in the structured interview as not present, subthreshold, or threshold. The clinician also rates whether the level of impairment is sufficient to qualify for DSM-5 diagnosis. MOODS-I was applied by trained psychiatrists in order to classify individuals by categorical diagnoses. Both instruments are available free of charge for research purposes upon request from the senior author (G.A.S.).

#### Electronic Chart Review Instrument

The chart review instrument^19^ was used to systematize diagnostic assessment for each participant from all of the outpatient programs participating in this study. Trained psychiatrists using the instrument reviewed a mean of 20 outpatient consultations (standard deviation [SD] = 9, range = 1-50), 0.6 inpatient admission notes (SD = 1.1, range = 0-8), and 0.57 inpatient discharge notes (SD = 1.02, range = 0-6). The trained psychiatrists used the instrument to systematically evaluate clinical charts in order to assess the patient diagnosis known a priori. The instrument assesses the following disorders: mood disorders (depression and mania), trauma-related disorders (post-traumatic stress disorder), anxiety disorders (specific phobia, social anxiety, panic disorder, agoraphobia, generalized anxiety disorder), obsessive-compulsive spectrum (obsessive-compulsive disorder), neurodevelopmental disorders (attention deficit/hyperactivity disorder), schizophrenia spectrum (schizophrenia, schizoaffective disorder), personality disorders (antisocial personality disorder, borderline personality disorder, histrionic personality disorder, narcissistic personality disorder) and substance-related disorders (alcohol abuse, drug abuse). The psychiatrist rates the likelihood of a diagnosis to be present or absent on a probabilistic scale as definitely absent, likely to be absent, likely to be present, or definitely present. For the purposes of this study, a diagnosis was considered to be present for likely to be present and definitely present. The instrument ensures a standardized method is used to collect information from electronic charts and has the advantage of taking a longitudinal perspective for patients using services for many years. This is particularly important for patients with severe mental disorders, because symptoms fluctuate over time.

#### Other symptom rating scales

The Hamilton Depression Rating Scale: This scale is administered by a clinical rater and contains 17 items measuring the severity of depressive symptoms over the last two months.^20 , 21^ The score ranges from 0 to 52, with higher scores representing greater depression severity. The scale’s coefficient of internal consistency is α = 0.75.^21^

The Young Mania Rating Scale (YMRS): This scale is also administered by a clinical rater, containing 11 items assessing the severity of manic symptoms over the last two months.^22 , 23^ The score ranges from 0 to 58, with higher scores representing greater severity of manic symptoms. The scale’s coefficient of internal consistency is α = 0.67.^23^

## Statistical analysis

We conducted CFA to evaluate a unidimensional model (with all items), a correlated model (with two dimensions, mood and behavior), and a bifactor model (with one general and two specific dimensions, mood and behavior). Correlations between CFA items were calculated with the mean and variance adjusted weighted least squares (WLSMV) estimator, implemented in the R (version 3.3.2) lavaan package (version 0.5-23).^24^ The model was considered to have a good fit to the data if the comparative fit index (CFI) and Tucker-Lewis index (TLI) were both ≥ 0.95 and the root mean square error of approximation (RMSEA) was ≤ 0.06. An acceptable fit to the data was defined as when fit indices CFI and TLI were ≥ 0.90, and RMSEA was ≤ 0.80.^25^ We also used Reykov omega coefficients as reliability indices.^26^ Item information curves were estimated from CFA using the R psych package.^27^ Path analysis was used to investigate associations with irritability-related impairment. Spearman correlation coefficients were used to investigate associations with clinical scales. Missing data were treated with imputation by chained equations using the mice package.^28 , 29^ Imputation accounted for 4% of the data.

## Results

### Sample description

The majority of our sample was composed of adult females from low to middle-income classes. One third of the sample had an anxiety disorder, two thirds had a mood disorder, and one-fourth met criteria for a psychotic disorder. Complete information on the sample can be found in Table 1 .

**Table 1:** Descriptive characteristics of study participants

	n (%) or mean ± SD
Age	44.56 ± 13.3
Male	93 (37.8)
SES	
A	5 (2.03)
B	72 (29.27)
C	127 (51.63)
D	23 (9.35)
Disruptive diagnosis	
DMDD	6 (2.44)
ODD	5 (2.03)
IED	20 (8.13)
Any anxiety disorder	101 (41.06)
Specific phobia	8 (3.25)
Social phobia	15 (6.10)
Panic	38 (15.45)
Agoraphobia	27 (10.98)
GAD	38 (15.45)
OCD	23 (9.35)
PTSD	26 (10.57)
Any mood disorder	197 (80.08)
Unipolar depression	190 (77.24)
Bipolar I	74 (30.08)
Bipolar II	92 (37.4)
ADHD	5 (2.03)
Any psychosis	61 (24.8)
Schizophrenia	52 (21.14)
Schizoaffective	13 (5.28)
Any disorder	241 (97.97)

ADHD = attention deficit hyperactivity disorder; DMDD = disruptive mood dysregulation disorder; GAD = generalized anxiety disorder; IED = intermittent explosive disorder; ODD = oppositional defiant disorder; PTSD = post-traumatic stress disorder; SD = standard deviation; SES = socioeconomic status (according to ABEP criteria^30^).

Sample recruited from outpatient psychiatry services at the Hospital de Clínicas de Porto Alegre (n = 246).

### Prevalence of irritability levels and disruptive disorders

Item frequencies for each component of the MOODS questionnaire are shown in Table 2 . Most of the participants showed low to moderate frequency of irritable mood and behavior symptoms. The most prevalent disruptive disorder diagnosis was IED with 8% prevalence, followed by DMDD with 2% and ODD with 2% prevalence.

**Table 2:** Mood-Disruptive Scale (MOODS) item frequencies on the seven-day scale

Irritable mood (item code/content)	Not at all	A little	Moderately	A lot	Extremely	
m3a						
Angry?	97 (39.4)	80 (32.5)	38 (15.4)	26 (10.5)	5 (2.0)	
m3b						
Touchy?	115 (46.7)	52 (21.1)	30 (12.2)	39 (15.8)	10 (4.0)	
m3c						
Grumpy?	77 (31.3)	92 (37.4)	22 (8.9)	39 (15.8)	16 (6.5)	
m3d						
Cranky?	99 (40.2)	71 (28.8)	27 (10.9)	30 (12.2)	19 (7.7)	
m3e						
Annoyed?	55 (22.3)	84 (34.1)	35 (14.2)	46(18.7)	26 (10.5)	
m3f						
Frustrated?	80 (32.5)	72 (29.2)	25 (10.1)	46 (18.7)	23 (9.3)	
m3g						
Resentful?	92 (37.4)	62 (25.2)	37 (15.0)	37 (15.0)	18 (7.3)	
m3h						
Surly?	123 (50.0)	62 (25.2)	26 (10.5)	25 (10.1)	10 (4.0)	
m3i						
Irritable?	94 (38.2)	67 (27.2)	32 (13.0)	29 (11.7)	24 (9.7)	
m3j						
Short-tempered?	114 (46.3)	56 (22.7)	24 (9.7)	29 (11.7)	23 (9.3)	

**Disruptive behavior (item code/content)**	**Never**	**Rarely**	**Some days of the week**	**Most days of the week**	**Every day of the week**	**Many times each day**

t2a						
Shouted?	103 (41.8)	77 (31.3)	39 (15.8)	11 (4.4)	7 (2.8)	9 (3.6)
t2b						
Took your anger out on objects?	170 (69.1)	48 (19.5)	19 (7.7)	4 (1.6)	2 (0.8)	3 (1.2)
t2c						
Threatened violence toward others?	188 (76.4)	38 (15.4)	15 (6.1)	2 (0.8)	1 (0.4)	2 (0.8)
t2d						
Threw things at someone?	214 (86.9)	15 (6.1)	12 (4.8)	2 (0.8)	0 (0)	3 (1.2)
t2e						
Cursed or swore aloud?	120 (48.7)	70 (28.4)	36 (14.6)	10 (4.0)	4 (1.6)	6 (2.4)
t2f						
Kicked furniture, walls or doors?	192 (78)	31 (12.6)	17 (6.9)	4 (1.6)	1 (0.4)	1 (0.4)
t2g						
Intimidated someone?	196 (79.6)	36 (14.6)	10 (4)	2 (0.8)	1 (0.4)	1 (0.4)
t2h						
Slammed doors?	170 (69.1)	47 (19.1)	17 (6.9)	10 (4.0)	1 (0.4)	1 (0.4)
t2i						
Pushed someone?	209 (84.9)	24 (9.7)	7 (2.8)	5 (2.0)	0 (0)	1 (0.4)
t2j						
Verbally insulted someone?	157 (63.8)	59 (23.9)	21 (8.5)	3 (1.2)	1 (0.4)	5 (2.0)
t2k						
Broke objects (e.g., TV, cell phone)?	214 (86.9)	23 (9.3)	6 (2.4)	0 (0)	0 (0)	3 (1.2)
t2l						
Got involved in fights causing mild injury?	225 (91.4)	15 (6.1)	6 (2.4)	0 (0)	0 (0)	0 (0)
t2m						
Smashed windows?	193 (78.4)	36 (14.6)	12 (4.8)	5 (2.0)	0 (0)	0 (0)
t2n						
Hit or injured animals?	219 (89)	20 (8.3)	6 (2.4)	0 (0)	0 (0)	1 (0.4)
t2o						
Got involved in fights causing serious injury?	240 (97.5)	5 (2.0)	1 (0.4)	0 (0)	0 (0)	0 (0)
t2p						
Scratched or pulled other person’s hair?	223 (90.6)	15 (6.1)	6 (2.4)	2 (0.8)	0 (0)	0 (0)
t2q						
Yelled at someone?	117 (47.5)	81 (32.9)	21 (8.5)	11 (4.4)	6 (2.4)	10 (4.0)

**Impairment (item code/content)**	**Not at all**	**A little**	**A lot**	**Extremely**		

i2a						
With romantic partners?	92 (37.4)	70 (28.4)	25 (10.1)	7 (2.8)		
i2b						
With family members?	104 (42.2)	79 (32.1)	48 (19.5)	13 (5.2)		
i2c						
With friends?	161 (65.4)	60 (24.3)	20 (8.1)	4 (1.6)		
i2d						
At work?	114 (46.3)	29 (11.7)	9 (3.6)	4 (1.6)		
i2e						
At school/university?	115 (46.7)	14 (5.6)	6 (2.4)	2 (0.8)		
i2f						
With the law?	203 (82.5)	25 (10.1)	9 (3.6)	9 (3.6)		
i2g						
With your finances?	141 (57.3)	50 (20.3)	37 (15)	18 (7.3)		
i2h						
With your life in general?	88 (35.7)	80 (32.5)	62 (25.2)	16 (6.5)		

Data presented as n (%).

Sample recruited from outpatient psychiatry services at the Hospital de Clínicas de Porto Alegre (n = 246).

### Confirmatory factor analysis

The CFA indices showed that the unidimensional model encompassing both mood and disruptive behavior did not have a good fit to the data (RMSEA = 0.146, 90% confidence of interest [90%CI] = 0.14-0.15; CFI = 0.96; TLI = 0.95). Omega reliability was 0.96.

**Figure 1:**
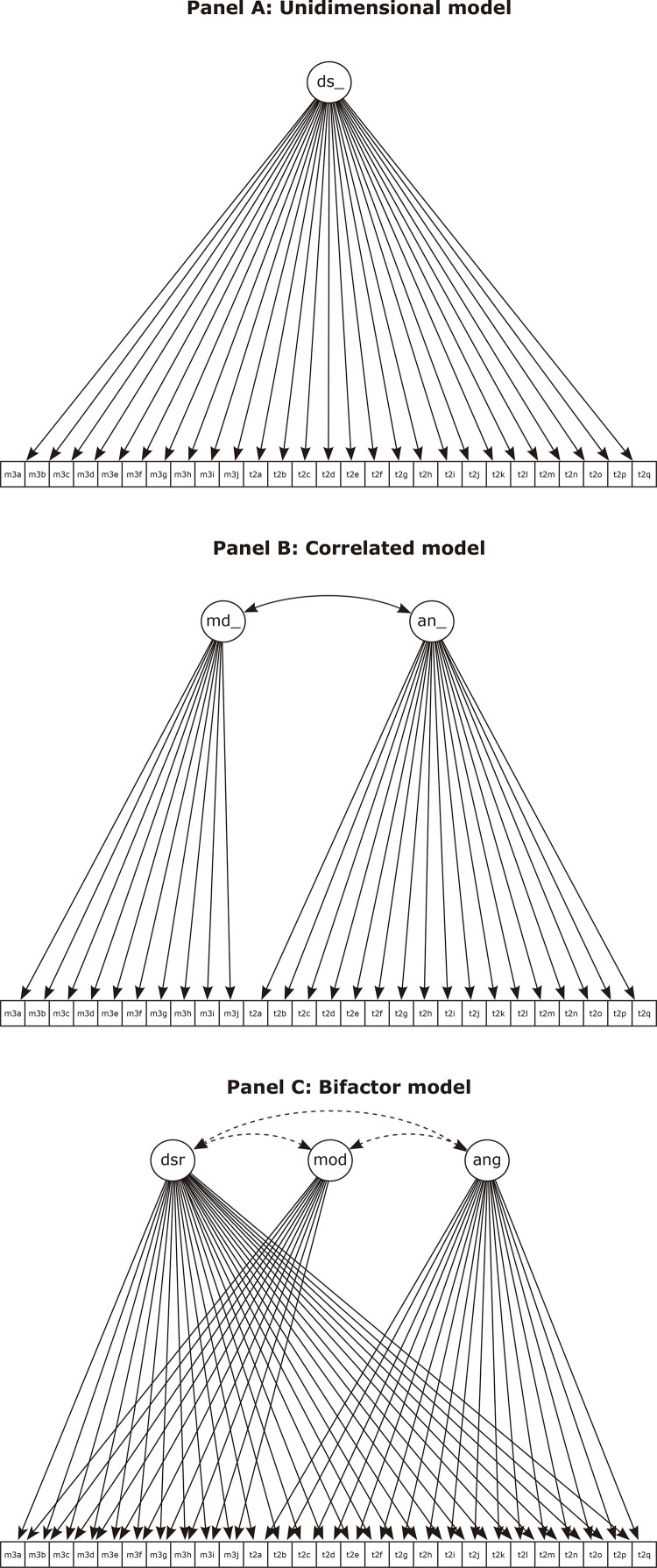
Sample recruited from outpatient psychiatry services at the Hospital de Clínicas de Porto Alegre (n = 246). Panel A illustrates the unidimensional model, Panel B shows the correlated model, and Panel C depicts the bifactorial model. an_ = disruptive behavior dimension; ang = disruptive behavior dimension; ds = general factor; dsr = general factor; md_ = irritable mood dimension; mod = irritable mood. Full descriptions of the abbreviations for each question (m3a-t2q) are given in Tables 2 and 3.

The correlated model (with two dimensions: mood and behavior) had acceptable fit indices (RMSEA = 0.077, 90%CI = 0.071-0.083; CFI = 0.99; TLI = 0.99) with high item loadings for the mood dimension (0.82-0.91; median = 0.86) and the behavior dimension (0.57-0.89; median = 0.73). Omega reliability for mood was 0.94 and for behavior it was 0.95. The unidimensional model, bifactor model, and correlated two factor model are illustrated in Figure 1 .

The bifactor model with a general dimension (common) and two specific dimensions (mood and behavior) had an excellent fit to the data (RMSEA = 0.041; CFI = 0.99; TLI = 0.99); with item loadings for the common dimension ranging from 0.21 to 0.90 (median = 0.55); from 0.16 to 0.66 (median = 0.41) for the mood dimension ; and from 0.24 to 0.94 (median = 0.59) for the behavior dimension. Omega reliability for the general factor was 0.61 and was 0.90 and 0.93 for the mood and behavior factors respectively. Analysis of item endorsement, as depicted by the category thresholds, showed that mood components of irritability were already present in patients with less severe irritability symptoms. Behavioral correlates, however, were only present in more severe cases. Furthermore, some of the variables in the bifactor model had low factor loadings (< 0.3). In our study, these variables, including m3a, m3b, m3h, m3j, and t2e, contributed less to the latent construct they should reflect. The CFA results are shown in Table 3 .

**Table 3:** Standardized factor loadings and category thresholds for unidimensional model, correlated model with two dimensions, and bifactor model

	Uni (λ)	Correlated (λ)		Bifactor (λ)			Item thresholds					

	Common	Mood	Behavior	Common	Mood	Behavior	t1	t2	t3	t4	t5	t6
Irritable mood (item code/content)												
m3a												
Angry?	0.80	0.84	-	0.83	0.16	-	-0.28	0.59	1.15	2.05	-	-
m3b												
Touchy?	0.84	0.88	-	0.84	0.24	-	-0.08	0.46	0.84	1.74	-	-
m3c												
Grumpy?	0.79	0.82	-	0.74	0.39	-	-0.49	0.49	0.76	1.51	-	-
m3d												
Cranky?	0.83	0.86	-	0.80	0.33	-	-0.24	0.51	0.86	1.42	-	-
m3e												
Annoyed?	0.87	0.88	-	0.66	0.66	-	-0.76	0.17	0.56	1.27	-	-
m3f												
Frustrated?	0.87	0.89	-	0.68	0.62	-	-0.45	0.31	0.58	1.32	-	-
m3g												
Resentful?	0.85	0.87	-	0.68	0.59	-	-0.32	0.32	0.75	1.4	-	-
m3h												
Surly?	0.85	0.88	-	0.84	0.27	-	0	0.67	1.07	1.74	-	-
m3i												
Irritable?	0.89	0.91	-	0.85	0.36	-	-0.3	0.4	0.79	1.30	-	-
m3j												
Short-tempered?	0.87	0.91	-	0.90	0.17	-	-0.09	0.49	0.80	1.32	-	-
Disruptive behavior (item code/content)												
t2a												
Shouted?	0.73	-	0.79	0.69	-	0.36	-0.28	0.59	1.15	2.05	-	-
t2b												
Took your anger out on objects?	0.71	-	0.76	0.61	-	0.47	0.50	1.19	1.70	2.05	2.25	-
t2c												
Threatened violence toward others?	0.84	-	0.87	0.67	-	0.59	0.72	1.40	2.05	2.25	2.40	-
t2d												
Threw things at someone?	0.72	-	0.76	0.59	-	0.51	1.11	1.48	2.14	2.40	-	-
t2e												
Cursed or swore aloud?	0.75	-	0.80	0.75	-	0.24	-0.03	0.75	1.40	1.74	1.97	-
t2f												
Kicked furniture, walls or doors?	0.86	-	0.89	0.69	-	0.59	0.77	1.32	1.97	2.40	2.65	-
t2g												
Intimidated someone?	0.77	-	0.80	0.65	-	0.49	0.83	1.58	2.14	2.40	2.65	-
t2h												
Slammed doors?	0.83	-	0.87	0.75	-	0.44	0.49	1.17	1.62	2.40	2.65	-
t2i												
Pushed someone?	0.84	-	0.88	0.68	-	0.57	1.03	1.62	2.05	2.65	-	-
t2j												
Verbally insulted someone?	0.81	-	0.85	0.78	-	0.32	0.35	1.15	1.79	1.97	2.05	-
t2k												
Broke objects (e.g., TV, cell phone)?	0.72	-	0.77	0.64	-	0.44	1.13	1.84	2.40	-	-	-
t2l												
Got involved in fights causing mild injury?	0.76	-	0.81	0.41	-	0.89	1.37	1.97	-	-	-	-
t2m												
Smashed windows?	0.78	-	0.82	0.72	-	0.39	0.79	1.51	2.05	-	-	-
t2n												
Hit or injured animals?	0.67	-	0.71	0.53	-	0.50	1.23	1.84	2.40	-	-	-
t2o												
Got involved in fights causing serious injury?	0.48	-	0.57	0.21	-	0.94	1.97	2.65	-	-	-	-
t2p												
Scratched or pulled other person’s hair?	0.84	-	0.87	0.64	-	0.60	1.32	1.84	2.40	-	-	-
t2q												
Yelled at someone?	0.79	-	0.83	0.74	-	0.35	-0.06	0.86	1.23	1.51	1.75	-
Model fit												
RMSEA	0.146	0.077			0.041							
RMSEA 90%CI	0.14-0.15	0.071-0.083		0.033-0.050								
CFI	0.96	0.99			0.997							
TLI	0.95	0.99			0.996							
Reliability												
ω (Raykov)	0.96	0.94	0.95	0.95	0.90	0.93						
ωH				0.61								
ωS					0.45	0.42						

90%CI = 90% confidence interval; λ = factor loadings; ω = omega coefficient; ωH = omega hierarchical; ωS = omega subscale; Bi = bidimensional; CFI = comparative fit index; RMSEA = root mean square error of approximation; TLI = Tucker-Lewis index; Uni = unidimensional.

Thresholds represent the amount of factor loading z-score needed for a person be more likely to endorse the next response category (from never to rarely, for example) than the previous one.

Sample recruited from outpatient psychiatry services at the Hospital de Clínicas de Porto Alegre (n = 246).

### Associations with irritability related impairment

In the bifactor model, only the common factor (b = 1.316, β = 0.718, standard error [SE] = 0.130, p < 0.001) and the mood component (b = 0.791, β = 0.432, SE = 0.103, p < 0.001) were associated with impairment, and no significant associations were detected for the behavioral component (b = -0.039, β = -0.021, SE = 0.102, p = 0.703).

### Associations with depression and mania

The bifactor model revealed that most of the associations were driven by the common factor (ρ = 0.444, p < 0.001 for the Hamilton Depression Rating Scale [HAMD]; ρ = 0.271, p < 0.001 for YMRS), although there were significant associations with the specific mood component (ρ = 0.497, p < 0.001 for HADS; ρ = 0.176, p = 0.01 for YMRS), but not with the specific behavioral component (ρ= -0.09, p = 0.203 for HADS; ρ = 0.00, p = 0.999 for YMRS).

## Discussion

Irritability is a ubiquitous, transdiagnostic trait that has been explored extensively in youth. However, to date, no research has explored the psychometric properties or external validity of irritable mood and behavioral outbursts in adults. Our goal was to investigate the independence of the mood and behavioral components of irritability in a sample of participants with severe mental disorders by conducting confirmatory factor analysis and evaluating associations with external validators. Research on psychiatric phenomenology, such as irritability, is essential to empirically test definitions of our classificatory systems, in order to aid researchers and clinicians to improve understanding of such symptoms. Further comprehension of irritability and its psychometric proprieties is key to advancing future research on the topic and to testing interventions for affected patients. Our results are as follows. First, using confirmatory factor analysis, we found that the latent variables explaining variance in mood vs. behavioral symptoms have both common and specific origins. Second, whereas mood items are endorsed in less severe cases of irritability, behavioral symptoms are endorsed in more severe cases of irritability, indicating a differential severity threshold. Third, impairment was more strongly associated with mood than behavior, and only mood items were associated with irritability-related impairment in bifactor models. Lastly, clinical measures of depression and mania were only associated with the general factor and the specific mood component, not with the specific behavior component.

First, our data show that many of the mood and behavioral symptoms of irritability are shared under a general dimension, meaning that mood aspects are commonly present in subjects with high levels of behavioral outbursts, and behavioral outbursts are commonly seen in those with high levels of irritable mood. Nevertheless, we also found that one single common factor was not able to explain all the variance in irritability symptoms and residual factors including mood and behavior were necessary to capture variance not explained by their common factor. Therefore, there might be both common and distinct causal pathways leading to mood and behavioral symptoms in adults; which is consistent with the existence of non-specific risk factors leading to pleiotropic symptomatic manifestations; but also with the existence of specific risk mechanisms affecting systems linked to affect and mood but not to behavior and vice versa.

Although research discriminating mood and behavior components of irritability in adults is lacking, preliminary research has begun to discriminate between distinct aspects of irritability in children.^9^ The pediatric literature has described irritability as having a tonic aspect, characterized by angry mood between temper outbursts, which does not necessarily have behavior manifestations; and a phasic aspect, characterized by temper outbursts or tantrums, which is not necessarily associated with persistent angry mood. Although the main distinction between phasic and tonic irritability in children relies on the duration of the manifestation (tonic lasting longer than phasic); operational methods for classifying tonic and phasic irritability are very reliant on the mood and behavior distinction; with mood representing proneness to persistently experience anger as an emotion, and behavior representing proneness to respond frequently with anger to frustration. Consistent with our findings of a shared variance between mood and behavior, this study also found that these two aspects of irritability are closely related.^9^

Second, another important finding from item level CFA analysis showed mood items were more frequently endorsed than behavioral items in this sample of adults. Therefore, persistent irritable mood is likely to capture variation in irritability levels in subjects not yet severely affected by irritability symptoms; whereas disruptive behaviors appears late in the process, characterizing subjects who already have very severe levels of irritability and are likely to have irritable mood. Of course, this hypothesis can only be confirmed with longitudinal studies. This is the opposite of what was concluded in the pediatric literature,^9^ where mood symptoms were less frequent and characterized as indicating a more severe illness than behavioral items. We hypothesize that the increase in behavioral control that occurs after adolescence might partially explain these findings.

This distinction between frequency of irritable mood in children and adults highlights an important discussion about the placement of adult irritable mood in our current classificatory manuals. Currently, the DSM-5 includes IED and ODD categories for diagnosing irritability, as a chief concern, in adults. The IED category has no mood component and the ODD category has a minor mood component among several other dimensions; whereas DSM-5 includes DMDD for children, which encompasses both mood and behavior. This potential bias of the adult literature towards seeing irritability solely as a behavior can also be observed in the diagnosis of major depression, in which irritable mood is seen as a core feature in children, but not in adults. Attempts to draw attention to irritability in adults as a subtype of depression have not being successful.^31 - 33^ Another unexplored possibility for understanding this phenomenon is to study the DMDD diagnosis in adults, which may provide an interesting alternative for capturing the mood component in this population. This is important, because irritability in children has been shown to predict anxiety and depression in adults,^15^ exemplifying its role as part of a pattern of heterotypic comorbidity among these disorders (e.g., irritability in children predicting dysthymia in adults). However, the absence of a DSM syndrome in adults that captures the mood aspect of irritability may have prevented the literature from also showing some degree of homotypic continuity (i.e., irritability in children predicting irritability in adults); a pattern which is also common for other emotional disorders such as anxiety and depression.^34^

Third, impairment was strongly associated with the shared variance between mood and behavior, but when looking into associations with specific factors, only the specific mood factor was associated with irritability-related impairment, while the specific behavioral factor was not. Results from the bifactor model are capable of disentangling shared from specific variance and reveal that all associations between behavior and impairment were shared with mood in the general factor, showing the prominence of irritable mood as leading to a range of problems in life. This also means that irritable mood adds information to the vast body of research involving reactive aggression in adults, providing some preliminary validity for the study of irritable mood in this population. However, we must take into consideration that the prevalence of severe irritability symptoms was not high in our sample and that this may explain why only the general factor and irritable mood were associated with impairment, since behavioral correlates of irritability were only endorsed in severe cases.

Lastly, associations between the specific mood component and clinical ratings of mood also provide some validity for the separation of mood and behavioral components of irritability. It is worth noting that the Hamilton Depression Rating Scale does not contain any irritability items and therefore correlations between these measures are not due to item sharing. Also, the Young Mania Rating Scale includes one item of episodic irritability, a core feature of mania, assessed by mood variations during the clinical interview. This is quite different from the concept of chronic irritability that is being discussed here, which sees irritability as a trait, a proneness to experiencing anger relative to others.

Our study has several limitations. First, our sample included outpatients with severe mental illness, with medical comorbidities, and taking a variety of medications. Further community studies are needed to properly assess irritability in adults. Second, clinical disorders were diagnosed using non-validated standardized instruments based on electronic records. Third, a large number of the patients recruited declined or were unable to complete the research protocol due to the exclusion criteria, which might decrease external validity. Fourth, since the frequency of irritable mood and disruptive behavior in our sample was not high, our findings may not be generalizable to clinical populations with high levels of irritability. Fifth, a somewhat limited sample size did not allow us to perform group comparisons. Nevertheless, this is the first study using a newly designed clinical instrument using psychometric and clinical validators to investigate the distinction between mood and behavioral components of irritability.

## Conclusions

This study concludes that there are both shared and potentially distinct aspects of irritability in adults that can be differentiated by a clinical interview, thus potentially facilitating specifically targeted treatments. Irritable mood is very common in individuals with severe mental disorders, and behavioral symptoms are present in more severe cases of irritability. These data provide some psychometric support for studying the shared and specific aspects of irritability in adults.
