# Supplementary material for: Differentiating irritable mood and disruptive behavior in adults
Source: Trends Psychiatry Psychother. 2020 Nov 17;42(4):375–86. doi: 10.1590/2237-6089-2019-0078 (PMC7879084; doi:10.1590/2237-6089-2019-0078)
Supplement: Supplementary file 1 [file 2238-0019-trends-42-04-0375-suppl01.pdf]

http://dx.doi.org/10.1590/2237-6089-2019-0078
Differentiating irritable mood and disruptive behavior in adults - Knackfuss AC et al.

Table S1 - Correlation matrix for CFA items

	t2a	t2b	t2c	t2d	t2e	t2f	t2g	t2h	t2i	t2j	t2k	t2l	t2m	t2n	t2o	t2p	t2p	m3a	m3b	m3c	m3d	m3e	m3f	m3g	m3h	m3i	m3j
t2a	-																										
t2b	-0.004	-																									
t2c	-0.005	0.401*	-																								
t2d	0.021	0.455*	0.381*	-																							
t2e	0.124	0.042	0.063	0.074	-																						
t2f	0.005	0.410*	-0.001	0.387*	0.017	-																					
t2g	-0.009	0.423*	-0.003	0.397*	-0.002	0.995*	-																				
t2h	0.028	0.281*	0.028	0.270*	0.093	0.724*	0.730*	-																			
t2i	-0.005	0.399*	0.003	0.370	0.089	0.517*	0.517*	0.366*	-																		
t2j	0.008	0.285*	0.008	0.264*	0.111	0.701*	0.701*	0.507*	0.369*	-																	
t2k	0.029	0.399*	0.008	0.558*	0.110	0.521*	0.527*	0.366*	0.498*	0.361*	-																
t2l	-0.011	0.402*	-0.004	0.379*	-0.034	0.995*	0.995*	0.710*	0.496*	0.702*	0.500*	-															
t2m	-0.002	0.286*	-0.001	0.263*	0.003	0.701*	0.709*	0.519*	0.360*	0.496*	0.364*	0.711*	-														
t2n	0.018	0.291*	-0.002	0.543*	-0.010	0.707*	0.702*	0.497*	0.362*	0.496*	0.724*	0.704*	0.493*	-													
t2o	-0.014	0.403*	-0.006	0.372*	-0.047	0.993*	0.994*	0.703*	0.496*	0.702*	0.501*	0.999*	0.704*	0.705*	-												
t2p	-0.010	0.402*	-0.004	0.374*	-0.024	0.995*	0.995*	0.709*	0.496*	0.724*	0.500*	0.998*	0.703*	0.703*	0.998*	-											
t2q	0.003	0.398*	-0.002	0.382*	0.078	0.986*	0.983*	0.727*	0.487*	0.731*	0.496*	0.982*	0.697*	0.694*	0.980*	0.984*	-										
m3a	0.015	-0.019	-0.008	0.203*	0.032	-0.002	-0.002	-0.007	-0.008	-0.007	-0.005	-0.001	-0.003	-0.002	-0.007	-0.001	0.016	-									
m3b	-0.004	-0.006	-0.008	-0.004	0.106	-0.008	-0.009	0.006	0.023	0.007	0.023	-0.014	0.004	0.002	-0.015	-0.012	-0.003	0.580*	-								
m3c	0.001	-0.010	-0.001	-0.011	0.044	-0.011	-0.013	0.004	0.015	0.009	0.009	-0.015	0.009	-0.014	-0.017	-0.013	-0.010	0.570*	0.030	-							
m3d	-0.008	-0.002	-0.005	-0.012	0.000	-0.012	-0.012	0.002	-0.002	0.002	0.006	-0.014	0.002	-0.009	-0.014	-0.012	-0.013	0.000	0.005	0.088	-						
m3e	-0.003	0.002	-0.005	-0.010	0.038	-0.005	-0.006	0.013	0.007	-0.002	0.008	-0.009	0.018	-0.012	-0.010	-0.008	0.007	-0.011	0.006	0.006	0.057	-					
m3f	0.010	0.002	-0.003	-0.018	0.152*	-0.013	-0.014	0.000	0.020	0.008	0.005	-0.018	-0.001	-0.017	-0.018	-0.017	-0.002	-0.003	0.038	0.006	0.007	0.021	-				
m3g	0.019	-0.003	0.002	-0.012	0.128*	0.003	-0.003	0.000	-0.008	0.007	0.001	-0.009	0.013	-0.012	-0.009	0.003	0.020	0.003	0.024	0.026	0.014	0.030	0.050	-			
m3h	0.015	0.003	0.013	-0.007	0.146*	-0.006	-0.008	0.009	0.002	0.016	0.011	-0.011	0.014	-0.002	-0.013	-0.009	0.016	0.001	0.021	0.009	0.013	0.016	0.038	0.037	-		
m3i	0.005	0.008	0.002	0.000	0.123	-0.008	-0.010	0.012	0.021	0.013	0.010	-0.015	0.010	-0.013	-0.016	-0.013	0.006	-0.010	0.002	0.001	0.020	0.011	0.040	0.028	0.034	-	
m3j	0.008	0.007	0.004	0.003	0.124	0.004	0.013	0.021	0.018	0.030	0.028	-0.004	0.019	0.009	0.005	0.000	0.020	-0.007	0.015	0.020	0.021	0.017	0.038	0.051	0.063	0.028	-

Sample recruited from outpatient psychiatry services at the Hospital de Clínicas de Porto Alegre (n = 246).
* $p < 0.05$.
